# The transcriptomes of cave and surface populations of *Gammarus minus* (Crustacea: Amphipoda) provide evidence for positive selection on cave downregulated transcripts

**DOI:** 10.1371/journal.pone.0186173

**Published:** 2017-10-09

**Authors:** David B. Carlini, Daniel W. Fong

**Affiliations:** Department of Biology, American University, Washington DC, United States of America; Institute of Crop Science, CHINA

## Abstract

*Gammarus minus*, a freshwater amphipod living in the cave and surface streams in the eastern USA, is an excellent model for investigating evolutionary adaptation to the subterranean environment. RNA-Seq was conducted on one pair of morphologically distinct sister populations inhabiting surface and cave habitats to identify genes that were differentially expressed in the two populations, as well as to compare levels and patterns of genetic variation within and between populations. Of the 104,630 transcripts identified in the transcriptome assembly, 57% had higher average levels of expression in the cave population. After Benjamini-Hochberg correction for multiple tests, 1517 and 551 transcripts were significantly upregulated or downregulated, respectively, in the cave population, indicating an almost three-fold enrichment of cave-upregulated genes. The average level of nucleotide diversity across all transcripts was significantly lower in the cave population. Within the cave population, where the average nucleotide diversity of cave-downregulated transcripts was 75% that of the cave-upregulated transcripts, a highly significant difference, whereas within the spring population the nucleotide diversities of cave-downregulated and cave-upregulated transcripts was virtually identical. Three lines of evidence suggest that the reduced variation in cave downregulated transcripts is due to positive selection in the cave population: 1) the average neutrality index of cave-downregulated genes was < 1, consistent with positive selection, and significantly less than that of cave-upregulated genes; 2) Tajima’s *D* was positively correlated with the cave:surface expression ratio, and 3) cave-downregulated transcripts were significantly more likely to be highly diverged from their surface homologs than cave-upregulated transcripts. Five transcripts had fixed premature termination codons in the cave population. The expression patterns and sequence variation in one such transcript, encoding the DNA repair protein photolyase, were examined in more detail and provide the first evidence for the relaxation of functional constraint in this light-dependent protein in a subterranean population.

## Introduction

To date, model organisms such as *Drosophila melanogaster* have provided the bulk of information that has contributed to our understanding of the molecular basis of adaptive change. However, data from non-model organisms, living in diverse habitats and expressing an array of interesting and potentially informative morphological adaptations, can provide a deeper knowledge of the evolutionary processes leading to adaptive morphological evolution. In particular, organisms which inhabit the subterranean realm provide a unique opportunity for studying adaptive, neutral, and/or regressive evolution due to the strong and consistent selective pressures associated with adapting to a novel environment characterized by complete darkness and low nutrient availability [1]. *Gammarus minus*, a freshwater amphipod crustacean living in both surface and subterranean habitats in the eastern United States, is an excellent candidate for studying the molecular basis of adaptive morphological change in a non-model organism [2,3].

Species that have adapted to subterranean habitats commonly possess morphological traits that arise via convergent evolution in the absence of light [4, 5]. These troglomorphic features often include regressive traits such as the reduction or total loss of eyes and body pigmentation, as well as constructive traits such as elaborated sensory organs (e.g., antennae), elongated appendages, and narrower bodies in comparison to their surface-dwelling sister taxa. The constructive traits, in particular, are generally considered to be adaptations to the dark arising from natural selection for enhanced extra-optic sensory perception and locomotive efficiency. The regressive features observed in subterranean species are a consequence of evolutionary loss, a phylogenetically widespread phenomenon [6]. Although constructive and regressive traits are not exclusive to subterranean taxa, both types of traits are frequently observed in the same subterranean species, in addition to having independently evolved in many unrelated taxa. The repeated independent evolution of such traits provides strong evidence that they are related to the common environmental condition, the complete absence of light, shared by subterranean taxa.

*G*. *minus* has an extensive geographic distribution in the carbonate rock formations of the eastern USA, extending southwesterly from Pennsylvania to Kentucky, Tennessee, and Southern Indiana. Within its range *G*. *minus* is a common inhabitant of carbonate springs characterized by hard, alkaline water of pH 6 or higher and conductivity greater than 100 μS/cm [7], where it is usually the dominant macroinvertebrate species in terms of density and biomass. Adults of spring populations have large compound eyes with about 40 ommatidia, a first pair of antennae at about 45–50% of body length, and brownish body pigmentation.

In addition to its surface spring populations, cave stream populations of *G*. *minus* are common throughout its geographic range. These caves streams resurge onto the surface at springs, and multiple lines of evidence provide support for the hypothesis that each of these cave populations have been independently founded by individuals from their hydrologically–connected upstream surface spring populations [2]. In most drainage basins, the morphology of individuals from cave and surface sister populations is virtually indistinguishable. However, morphologically divergent cave and surface populations of *G*. *minus* exist in two regions, 1) the Greenbrier Valley of southeastern West Virginia, and 2) the Wards Cove area of southwestern Virginia, where the cave systems in both regions are particularly extensive and large, with passages exceeding 20 km in length. In these two regions individuals from the cave populations possess classic troglomorphic adaptations, including larger body lengths, loss of or altered pigmentation, long antennae (≥ 65% of body length), and a pronounced reduction or complete loss of ommatidia [8].

*G*. *minus* is an excellent model organism in which to study adaptive evolution for several reasons. First, cave populations show heritable, individual variation in troglomorphic traits such as reduced eyes, reduced pigmentation, as well as the elaborated sensory structures [9]. Whereas, in contrast, for most other troglomorphic species all individuals lack any manifestation of eye and body pigment. Second, *G*. *minus* is a single species that contains populations that occupy both subterranean and surface habitats. This makes it a particularly useful model because it avoids the problem of inappropriate comparisons between distantly related species, as most troglomorphic species are highly endemic with no close surface relatives [10]. Third, hydrological and genetic evidence clearly indicate that troglomorphic populations of *G*. *minus* have repeatedly invaded cave streams from surface spring habitats, thus showing the troglomorphic traits are the derived condition [4]. Finally, evidence from allozyme variation [11] and from molecular sequence data [12] strongly indicates that multiple troglomorphic populations have each evolved troglomorphy independently from the non-troglomorphic state of their surface sister population. Given the existence of natural replicate cave and surface sister populations, it will be possible to see if the same genes are the targets of selection as exhibited through concordant patterns of expression and/or variation.

Only three other aquatic organisms in the world share this attribute with *G*. *minus*: consisting of multiple troglomorphic, subterranean populations and multiple non-troglomrophic, surface populations all belonging to the same species. They are the isopod crustacean *Asellus aquaticus* in Europe, the cave characin fish *Astyanax mexicanus* in Mexico [4], and the poeciliid fish *Poecilia mexicana* in Mexico [13]. We choose to work with *G*. *minus* because we have extensive experience with this species and we have access to all of the morphologically divergent spring and cave sister populations. The focus of this study is on one such pair of sister populations, a subterranean population from Organ Cave (OC) and a surface population from Ward Spring (WS), in the Second Creek Drainage Basin of Greenbrier Valley, WV. These two populations are hydrologically connected and proximate (< 3.5 km), and exhibit low levels of average pairwise nucleotide sequence divergence for both mitochondrial (< 1.5%, COI) and nuclear loci (< 0.5%, ITS-1, opsin) [12,14]. We present here the results of a descriptive study based on transcriptome profiling performed on multiple individuals from each of the two populations, comparing gene expression and nucleotide sequence variation within and between the populations.

## Methods

### Specimen collection and RNA isolation

Adult *G*. *minus* specimens were collected from Organ Cave (OC, 37.72°N, 80.44°W) and Ward Spring (WS, 37.69°N, 80.48°W) in Greenbrier County, West Virginia (USA) and transported live back to the laboratory, where they were maintained in commercial spring water (Deer Park) at 4°C in darkness. Specimens from OC were exposed to light only briefly, from illumination by a single LED headlamp during collection, exposure to tree-filtered daylight while transferring from collector’s cave packs to insulated coolers in vehicles, and exposure to fluorescent room light while transferring from coolers to incubators in the laboratory, for a total duration of less than 10 minutes. In addition, troglomorphic populations of *G*. *minus* did not react behaviorally to light *in situ* and were photo-neutral in the laboratory [15], probably because they have lost the ability to detect light or photo-avoidance behavior after detecting light. The collection and acclimation procedure did expose the individuals from the WS population to total darkness for a week, which is different from their natural daily photoperiod. However, surface *G*. *minus* were nocturnal *in situ*, being much more active at night and mostly hidden under rocks or vegetation during the day, as well as being photo-phobic in the laboratory [15]. Therefore, we are confident that our collection method has not significantly affected the physiological state of specimens from the OC and WS populations. After acclimating to the laboratory for a week, we chose two adult males and two adult females from each of the two populations for analysis. Surface-dwelling *G*. *minus* usually mature in 9–12 months from birth and die during their second year of life [16], while such life-history information is unknown for troglomophic *G*. *minus* populations. In the laboratory, individuals from both surface and troglomorphic cave populations live for three years or less. Thus we assume that all eight individuals used in this study were about one-year old.

After acclimation, individuals were transferred to vessels containing 100μg/mL ampicillin and 50μg/mL kanamycin in spring water for a week to minimize bacterial RNA contamination. Animals were not fed during the antibiotic treatment, and the water was changed and fresh antibiotic added once midway through the one-week treatment period. RNA extractions were performed on whole individuals using the RNeasy Plus Universal Kit (Qiagen), which contains a gDNA elimination solution to eliminate genomic DNA. RNA was quantified on a NanoVue spectrophotometer and RNA integrity was determined through capillary electrophoresis.

### RNA-seq library construction, sequencing, and de novo transcriptome assembly

Individual RNA libraries were constructed using the TruSeq^®^ RNA Sample Preparation Kit (Illumina, Inc.) using total RNA from each specimen. Briefly, the TruSeq^®^ RNA library construction protocol involved the purification of poly(A)-containing mRNA molecules from total RNA using oligo-dT attached magnetic beads, followed by RNA fragmentation, cDNA synthesis, adaptor ligation, and the addition of unique indexing barcodes to each library. Paired-end 2x100 sequencing of RNA libraries was performed on the Illumina^®^ HiSeq^™^ 2000 next-generation sequencing system.

A total of 295,740,592 post-filter high quality paired-end 100 bp reads were obtained from the eight *G*. *minus* specimens sequenced on the Illumina HiSeq2000 platform (Table 1). The number of high-quality reads per specimen ranged between 39,100,000 and 47,620,000, with no significant difference between the average number of reads per cave and surface specimens (43,759,615 versus 40,231,982 respectively, *P* = 0.12). Average Phred quality scores (*Q*) were very similar from specimen to specimen (34.91 ≤ *Q* ≤ 35.38), with approximately 90% of reads having *Q* values ≥ 30.

**Table 1:** Summary of Illumina sequencing statistics *Gammarus minus* specimens collected from Organ Cave (OC) and Ward Spring (WS).

Sample	Sex	Raw reads	Mapped reads	Unmapped reads	% Reads Q≥30	Mean Q Score
OCF1	F	40,225,792	33,714,763	6,511,029	89.44	34.92
OCF2	F	47,621,752	40,300,676	7,321,076	91.02	35.38
OCM1	M	45,293,636	37,661,063	7,632,573	89.49	34.93
OCM2	M	41,897,278	34,834,965	7,062,313	89.65	34.97
WSF1	F	41,546,462	33,594,500	7,951,962	89.86	35.04
WSF2	F	39,100,526	29,494,826	9,605,700	89.61	34.97
WSM1	M	39,534,272	31,639,595	7,894,677	90.71	35.30
WSM2	M	40,746,666	32,143,514	8,603,152	89.41	34.91

The reads from all eight samples were deposited at the NCBI Short Read Archive (SRA) Database (Accession numbers SRX2834574 –SRX2834581) and were used to build a consensus *de novo* transcriptome assembly using the Trinity software package [17] following a previously published protocol [18] with the minimum contig length set to 200 bp. The *de novo* transcriptome assembly yielded 104,630 putative transcripts. The median, average, and N50 contig lengths were 520, 1363, and 3241 bp, respectively. The proportion of reads from each specimen that mapped to the assembled transcriptome ranged between 75.4 and 84.6%. The 104,630 transcripts comprised 37,567 unique components (herein referred to as unigenes), yielding an average of 2.79 transcripts per gene. The longest transcript for each unigene was used in subsequent analyses of unigenes (e.g., expression level, gene ontology, polymorphism).

### Homology, gene ontology, expression, and variation

Blastx searches against the NCBI protein sequence database were performed for each transcript and the top 10 hit with *E*-values < 10^−3^ were retained [19]. For each unigene, the alignment of the top hit was used to determine the reading frame for subsequent analyses. Biological Process gene ontologies were obtained for each top hit via the Blast2GO server [20].

Read counts of each transcript from each specimen were obtained using the RNA Sequence Expectation-Maximization (RSEM v1.2) software package [21] and normalized by dividing by the total number of reads in each specimen to obtain a standardized measure of expression, reads per million mapped reads (RPM). In order to confirm that the read counts obtained using RSEM were reproducible using a different quantification method, we also obtained read counts of each transcript from each specimen using the recently developed kallisto RNA-seq quantification program, which has been shown to be an efficient and rapid method for quantifying transcript abundances [22]. For the entire set of 104,630 transcripts the correlation coefficient (Pearson’s *r*) between RSEM and kallisto derived transcript abundances in the OC population was 0.97, while the correlation coefficient for WS population was 0.98. Henceforth, all analyses and results are based on the RSEM derived transcript abundances. Differentially expressed genes were identified by performing Welch’s *t*-tests on cave versus surface RPM values using the Benjamini-Hochberg procedure to control for the false discovery rate in conducting multiple comparisons [23]. To check for congruence, differential expression analysis was also conducted using the Bioconductor software package edgeR [24], which models the variability of read count data based on the negative binomial distribution, again with a Benjamini-Hochberg correction for multiple comparisons. Differentially expressed transcripts derived from Welch *t*-tests and edgeR analysis were in strong agreement, as discussed below.

Variants were called using the samtools 1.2 [25]. The Genome Analysis Tool Kit (GATK) utility HaplotypeCaller was used to filter variants with low quality scores using variant quality score recalibration following the protocol outlined in the GATK best practice pipeline to produce high confidence variant calls [26]. The GATK utility FastaAlternateReferenceMaker was employed to generate fasta files for each transcript in each sample, incorporating variant sites obtained from the output of HaplotypeCaller [27].

BioPerl modules were used to conduct pairwise sequence comparisons and calculate Tajima’s *D* [28]. Perl scripts were written to quantify synonymous and nonsynonymous polymorphisms and fixations required to compute the neutrality index [29] and its derivatives [30] for unigenes with significant blast hits and alignment lengths of at least 100 codons, as well as to identify premature termination codons. Briefly, for those unigenes with blast hits the reading frame and sequence coordinates were obtained from the blastx output and used to align the eight GATK-generated fasta files corresponding to each transcript. The eight sequences were then compared at each codon position to identify premature termination codons in one or more transcripts, as well as to quantify the number of 1) synonymous polymorphisms (*P*_*S*_), synonymous polymorphisms within OC or WS, or within both, 2) nonsynonymous polymorphisms (*P*_*N*_), nonsynonymous polymorphisms within OC or WS, or within both, 3) synonymous fixations (*F*_*S*_), fixed synonymous differences between OC and WS, and, 4) nonsynonymous fixations (*F*_*N*_), fixed nonsynonymous differences between OC and WS).

## Results and discussion

### Expression and homology

**Fig 1:**
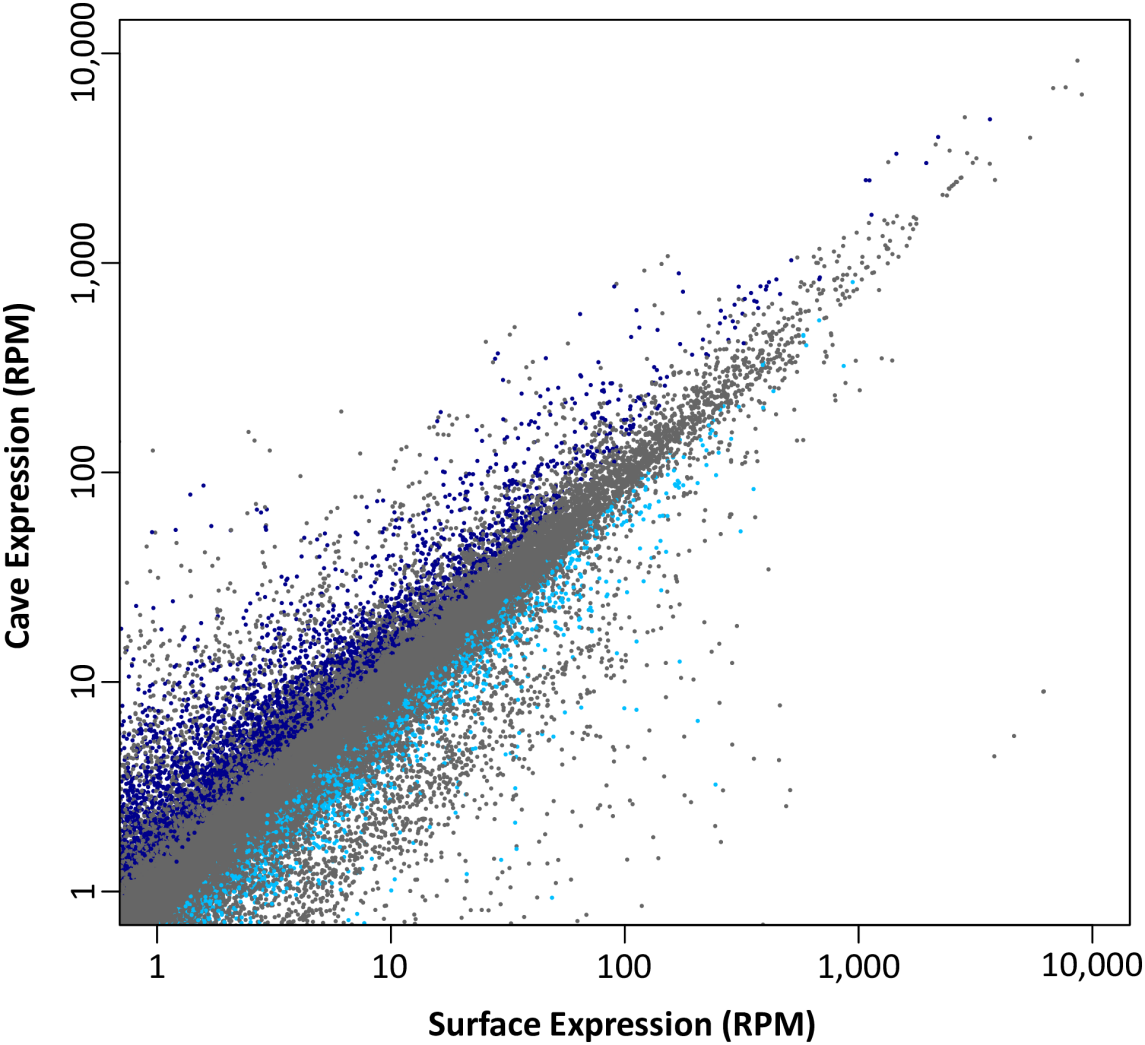
Scatterplot of the averages of standardized expression levels (RPM = reads per million mapped reads) in four cave versus four surface specimens for all 104,630 transcripts in the transcriptome assembly (R^2^ = 0.85). A greater number of transcripts had higher expression in the cave population than in the surface population (59,571 versus 45,069, respectively). Data points from transcripts with significantly different levels of expression in the cave and surface populations are labeled in color (dark blue = cave upregulated, light blue = cave downregulated).

The average standardized expression levels (reads per million mapped reads) in the four cave and four surface samples was calculated for each transcript (Fig 1), resulting in 59,571 (57%) cave–upregulated (CU) and 45,059 (43%) cave–downregulated (CD) transcripts, a highly significant departure from an expectation of an equal proportion of CU and CD transcripts (chi-square = 1006.4, *P* < 10^−100^). Although it is possible to categorize each transcript (or component) as CU or CD simply based on the average expression levels in the cave and surface populations, the majority of transcripts were not significantly differentially regulated between the cave and surface populations. After Benjamini-Hochberg adjustment for multiple comparisons, 2068 (~2%) transcripts were identified through Welch’s *t*-tests as differentially regulated, with 1517 (73%) of them CU transcripts and 551 (27%) of them CD transcripts, mirroring the enrichment of CU transcripts observed for the entire set of 104,630 transcripts. Results from edgeR analysis of differential expression were congruent with Welch’s t-test results. All 1517 CU transcripts were also upregulated in the cave population using edgeR, and 1348 of the 1517 (89%) were also identified as significantly differentially regulated in edgeR. All 551 CD transcripts were also downregulated in the cave population in the edgeR analysis, and 523 of the 551 (95%) were also identified as significantly differentially regulated between OC and WS using edgeR.

The average expression levels of unigenes exhibited nearly the same bias toward CU transcripts, with 21,662 (58%) of the unigenes CU and 15,905 (42%) CD, a highly significant difference from a null expectation of equal proportions of CU and CD transcripts (chi-square = 882.2, *P* < 10^−100^). After correction for multiple tests, 1,211 of the 37,567 unigenes were significantly differentially expressed, with 826 (68%) CU unigenes, and 385 (32%) CD unigenes. As was the case for the entire set of transcripts, the skew toward cave upregulation for the 1211 differentially expressed unigenes was highly significant (chi-square = 210.2, *P* < 10^−46^).

To our knowledge this is the first evidence of a general tendency toward upregulation of gene expression in a subterranean population or species. In general, previous studies have documented differences in the expression of sets of related genes, such as a reduction in the expression of genes related to vision or circadian rhythms in subterranean populations or species. For example, a study of circadian rhythms in Mexican blind cavefish (*Astyanax mexicanus*) demonstrated the upregulation of three light-inducible genes (*period 2*: a circadian rhythm gene, *CPD photolyase*: a photoreactivation DNA repair gene, and damage-specific DNA binding protein 2: a nucleotide excision DNA repair gene) in cave populations relative to their surface counterparts [31]. A transcriptome profiling study on eye samples from a blind cavefish (*Sinocyclocheilus anophthalmus*) and a closely related surface congener (*S*. *angustiporus*) revealed a general tendency toward decreased expression in the cave population [32]. Of the differentially regulated genes (> twofold change in expression), 71.7% exhibited decreased expression in the cave species. Similarly, significantly decreased expression levels of three different opsin transcripts were observed in multiple subterranean diving beetle species in comparison with related surface species [33]. We also observed significant decreases in the expression of visual genes in the subterranean population (discussed below), but the general trend toward upregulation in the cave population is difficult to explain. One possibility is that the one-week starvation treatment that specimens were subject to prior to RNA extractions to clear their guts of microbes had a more pronounced effect on the individuals from the WS surface population. Because food is a scarcer resource in the cave environment, it may be that specimens from the OC population were better adapted to the starvation treatment and consequently were able to maintain normal levels of transcription, whereas starvation adversely affected metabolism and homeostasis in the specimens from the WS population, resulting in a net loss of transcription globally. Another possibility is that the same antibiotic treatment caused a difference in response in terms of overall expression between the two populations. Gene expression response to antibiotic treatment is well documented in bacteria and eukaryotic cell cultures [34–35], however, we know of no study linking antibiotic treatment to differential responses in terms of global changes in gene expression in whole animal crustaceans. Furthermore, as we detail below, lowered expression levels of genes related to vision in the OC compared to WS populations in this study were consistent with the difference in habitat and consistent with results from previous studies. Therefore, we are confident that effects from antibiotic treatment on global expression levels was minimal.

**Fig 2:**
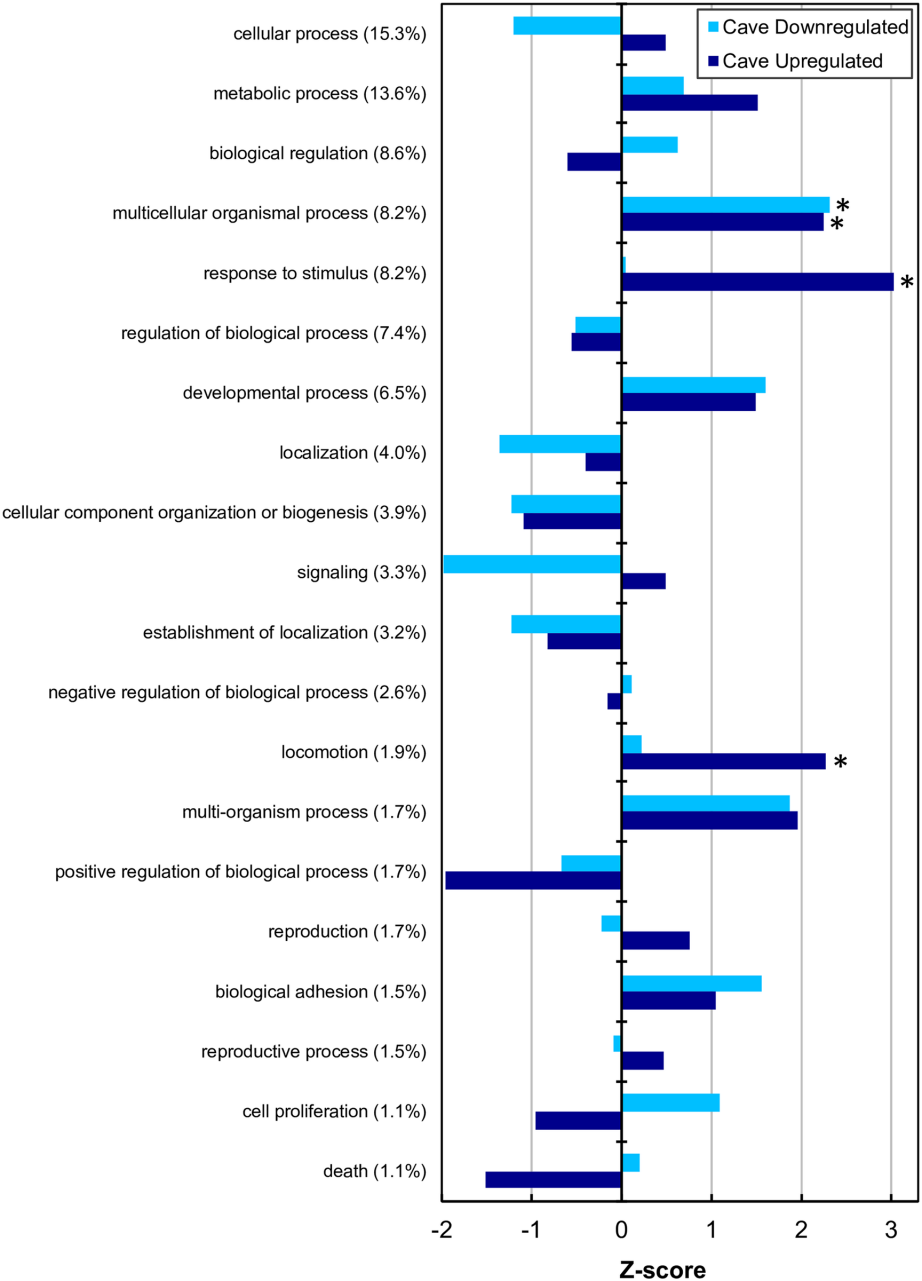
Z-scores of top 20 biological process gene ontologies for significantly differentially regulated unigenes in comparison with the entire set of 8,134 unigenes. None of the gene ontologies were significantly underrepresented (Z score > 2) for either cave downregulated (dark blue) or cave upregulated (light blue) unigenes, and three gene ontologies, indicated by asterisks, were significantly enriched: multicellular organismal process for both cave downregulated and cave upregulated unigenes, response to stimulus for cave upregulated unigenes, and locomotion for cave upregulated unigenes.

Blast hits were obtained for 25,856 of the 104,630 transcripts (24.7%), and for 8,134 of the 37,567 unigenes (21.7%). Overall, of the 25,856 top hits, 95% had an E value < 10^−4^, and 75% had an E value < 10^−8^. Of the 1,211 differentially expressed unigenes, 720 (59.5%) had a Blast hit. The most common Biological Process gene ontologies were cellular process (15.3%) and metabolic process (13.6%), followed by biological regulation (8.6%), multicellular organismal process (8.2%), response to stimulus (8.2%), regulation of biological process (7.4%), and developmental process (6.5%). The remaining ontology categories accounted for less than 5% of the Blast hits. For both CD and CU unigenes, approximately half (9 of 20 each) were underrepresented and half (11 of 20) were enriched relative to the entire set of 8,134 unigenes with a Blast hit. For most Biological Process GO terms, the degree of underrepresentation or enrichment was not significant (|*z*-score| < 2), but three GO terms were significantly enriched: multicellular organismal process in CD and CU unigenes, response to stimulus in CU unigenes, and locomotion in CU unigenes (Fig 2). Of the 20 top GO terms, seven were enriched in both CD and CU unigenes, and five were underrepresented in both CD and CU unigenes. Among the eight GO terms with divergent patterns of enrichment/underrepresentation, four were enriched in CD/underrepresented in CU unigenes, and four were underrepresented in CD/enriched in CU unigenes.

Notable transcripts that were significantly downregulated in the OC population included homologs of visually-related genes such as opsin (0.23×, *P* < 0.01), arrestin (0.15×, *P* < 0.01), and the Bardet-Biel syndrome protein (0.41×, P < 0.01). We previously reported the downregulation of two opsin homologs in the OC population (0.16× and 0.41×, respectively) [14], as well as the downregulation of *hedgehog* expression in the OC population relative to the WS population [36]. Although our results indicated a downregulation of *hedgehog*, a developmental gene that contributes to variation in eye size in *Drosophila* [37], the downregulation in OC was not statistically significant. We were unable to find any obvious candidates for significantly downregulated genes involved in pigmentation pathways (Biological Process GO term “Pigmentation”, GO ID “GO:0043473”).

### Polymorphism and tests for selection on CD unigenes

**Fig 3:**
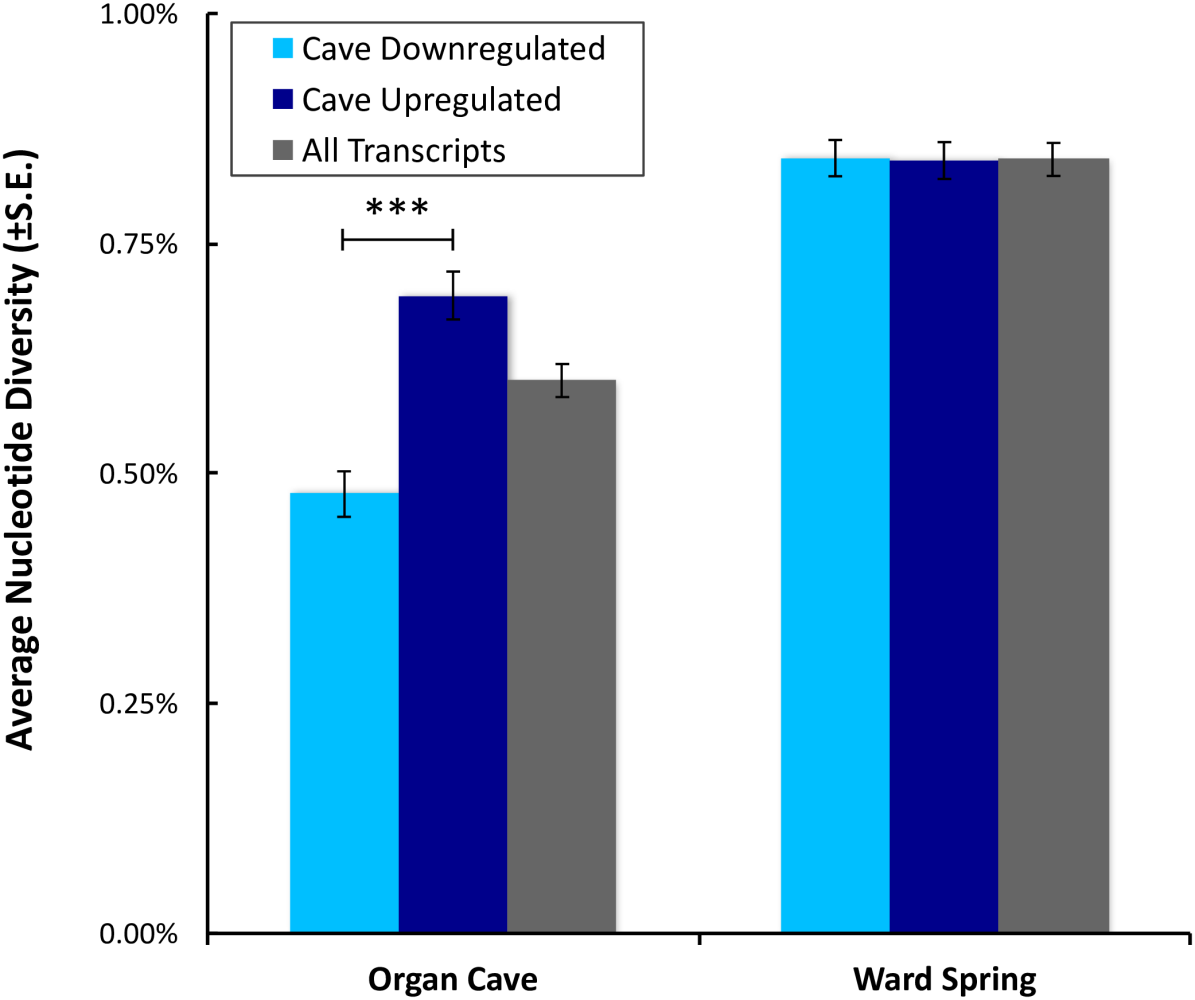
Average nucleotide diversities *(θ*_*p*_) of cave downregulated (rust), cave upregulated (dark blue), and all unigene transcripts (grey) within the Organ Cave (light blue) and Ward Spring (right) populations. In Ward Spring, the average nucleotide diversities of cave downregulated and cave upregulated genes were nearly identical at 0.840% and 0.843%, respectively. In Organ Cave, the nucleotide diversity of cave downregulated genes, 0.479%, was significantly less than that of cave upregulated genes, 0.693% (*P* < 10^−25^ Welch’s t-test, *P* < 10^−118^, paired t-test, as indicated by the asterisks).

The average level of nucleotide diversity (θ_π_) within the unigenes of the OC population was 0.601%, significantly less than that of the WS population, 0.841% (Fig 3, *P* < 10^−25^ Welch’s t-test, *P* < 10^−118^, paired t-test), consistent with previous studies in which levels of polymorphism in these two populations were determined [11,12]. Comparison of average nucleotide diversity in the CU versus CD unigenes within the OC population revealed an interesting and unexpected pattern, where the average nucleotide diversity of the CD transcripts (0.479%) was significantly less than that of the CU transcripts (0.693%) (*P* < 10^−9^, Welch’s t-test). It is unlikely that these differences in nucleotide diversity are an artifact of differences in read depth since the average read depth of the CD genes in the OC population, 13.1±43.2 RPM, was not significantly different from the average read depth of the CU genes in the WS population, 22.4±203.6 RPM (*P* > 0.05, Welch’s t-test). Thus, if these differences were an artifact of decreased read depth one would expect to see a mirrored pattern in the WS population, with the low read depth CU genes harboring significantly less nucleotide diversity than the high read depth CD genes in that population. However, this was clearly not the case within WS, where levels of nucleotide diversity were nearly identical in the CU and CD transcripts, 0.840% versus 0.843%, respectively (*P* = 0.92, Welch’s t-test).

A number of factors might be responsible for the nearly 1.5-fold reduction in levels of genetic variation for the CD transcripts in the OC population. First, it may be that the CD transcripts are subject to stronger purifying selection in the cave. If this were the case, then the neutrality index (NI), the odds ratio of fixed nonsynonymous to fixed synonymous substitutions (F_N_/F_S_) over nonsynonymous to synonymous polymorphisms (P_N_/P_S_) should differ between CD and CU transcripts [29]. NI values of one are consistent with neutral evolution, NI values less than one are indicative of positive selection, and NI values greater than one are consistent with purifying selection. For those significantly differentially regulated transcripts (raw *P* < 0.05) with defined NI values, the average NI of CD transcripts was 0.84, whereas the average NI of CU transcripts was significantly greater at 1.61 (P < 0.05, Welch’s t-test). These results indicate that, in general, CU transcripts are subject to stronger purifying selection than CD transcripts, opposite the expectation if reduced polymorphism in CD transcripts is due to purifying selection. However, since NI is a ratio of ratios it is undefined when F_S_, P_N_, or P_S_ are equal to zero, a common occurrence for intraspecific comparisons; consequently, a large fraction (>90%) of the differentially regulated transcripts were not included in the above NI comparisons. In such cases a log transformation of the NI, LNI_Laplace_ = Log [(F_S_+1)(P_N_+1)/(F_N_+1)(P_S_+1)], can be applied so that transcripts with undefined NI ratios are not discounted [30, 38]. Negative LNI_Laplace_ values indicate positive selection, positive LNI_Laplace_ values indicate purifying selection, and LNI_Laplace_ values of zero indicate neutrality. The average LNI_Laplace_ values of significantly CD transcripts was –0.14, significantly less than the average of CU transcripts, –0.04 (*P* < 0.05, Welch’s t-test). This is also consistent with CD transcripts having been subject to positive rather than purifying selection. An alternate explanation of the reduced variation in the CD transcripts of the OC population is that, on average, the regulatory regions of CD genes were subjected to stronger positive selection than the regulatory regions of CU genes in the OC population. In this case, the coding sequences themselves may not have been the targets of selection, rather, changes in the regulatory regions that altered expression levels were the targets of selection.

**Fig 4:**
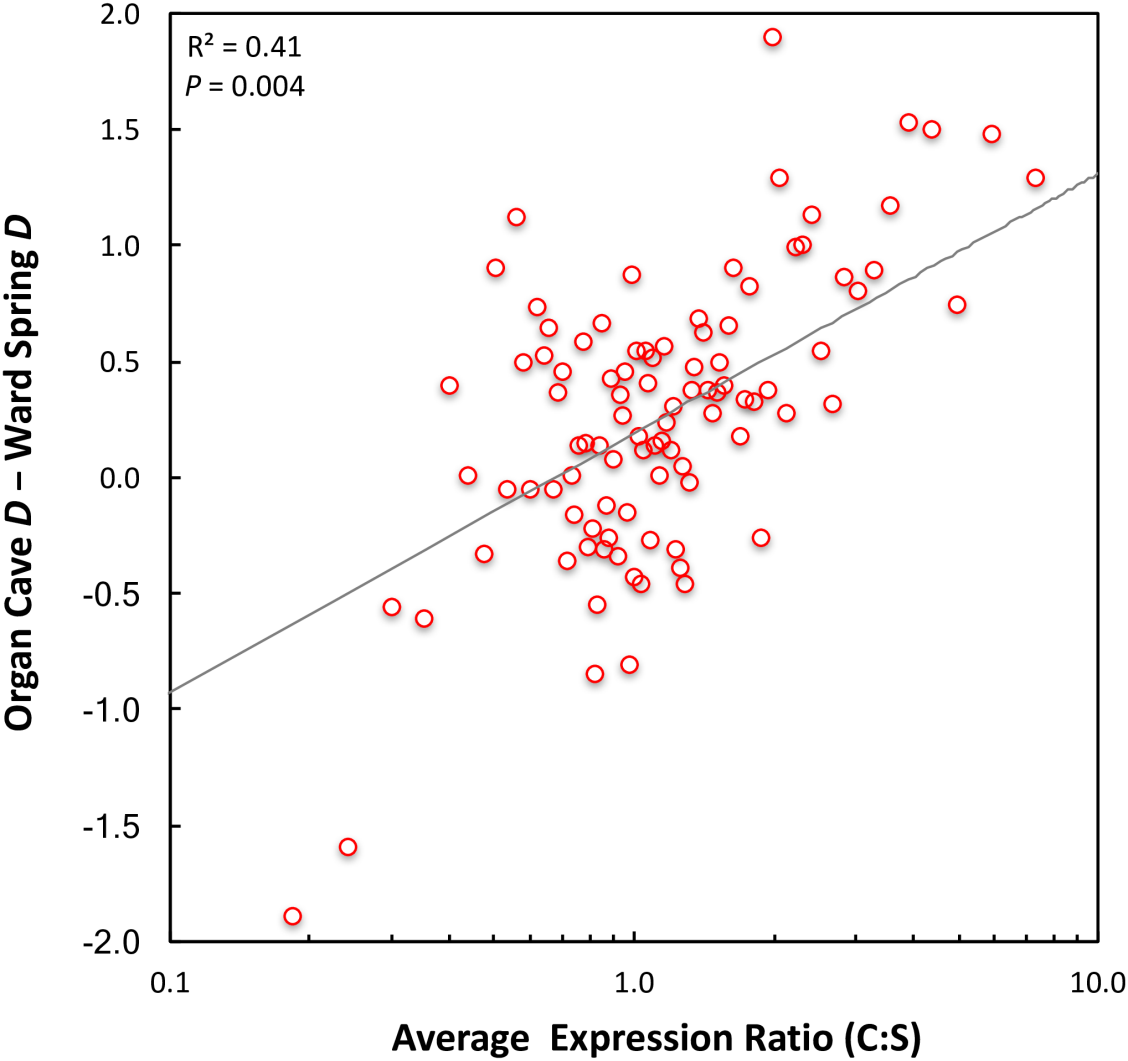
Difference in Tajima’s *D* (Organ Cave—Ward Spring) versus average expression ratio for binned data. Expression data for the 37,567 unigenes were binned in 1% intervals (~376 unigenes per bin) according to their cave:surface expression ratios. Each of the 100 data points in the plot consists of the average expression ratio in the bin (X-axis, log_10_ scale), and the average difference in Tajima’s *D* (y-axis). The least squares regression is shown in grey, had a coefficient of determination of 0.42 and the slope of the regression line is significantly positive (*P* < 0.01), indicating a positive relationship between Δ*D* and average expression ratio.

Two other approaches were used to determine if the CD transcripts were subject to positive selection in the OC population. First, Tajima’s *D* was calculated for each transcript [39]. Because there were only four sequences available from each population for each transcript, values of Tajima’s *D* are expected to be inflated due to a small sample size [40]. However, since sample sizes are equal in the two populations, Tajima’s *D* should be equally inflated in the two populations, and therefore the difference in Tajima’s *D* could be used to determine if the level of Tajima’s *D* was biased in one direction or the other as a function of expression ratio. Transcripts were binned into 1% expression ratio intervals, and a significant positive relationship between expression ratio and the difference in Tajima’s *D* (*D*_OC_−*D*_WS_) was obtained (Fig 4, *P* < 0.01). This relationship is consistent with expectations if, in general, the CD transcripts in the OC population were subjected to stronger positive selection than CD transcripts in the WS population.

**Fig 5:**
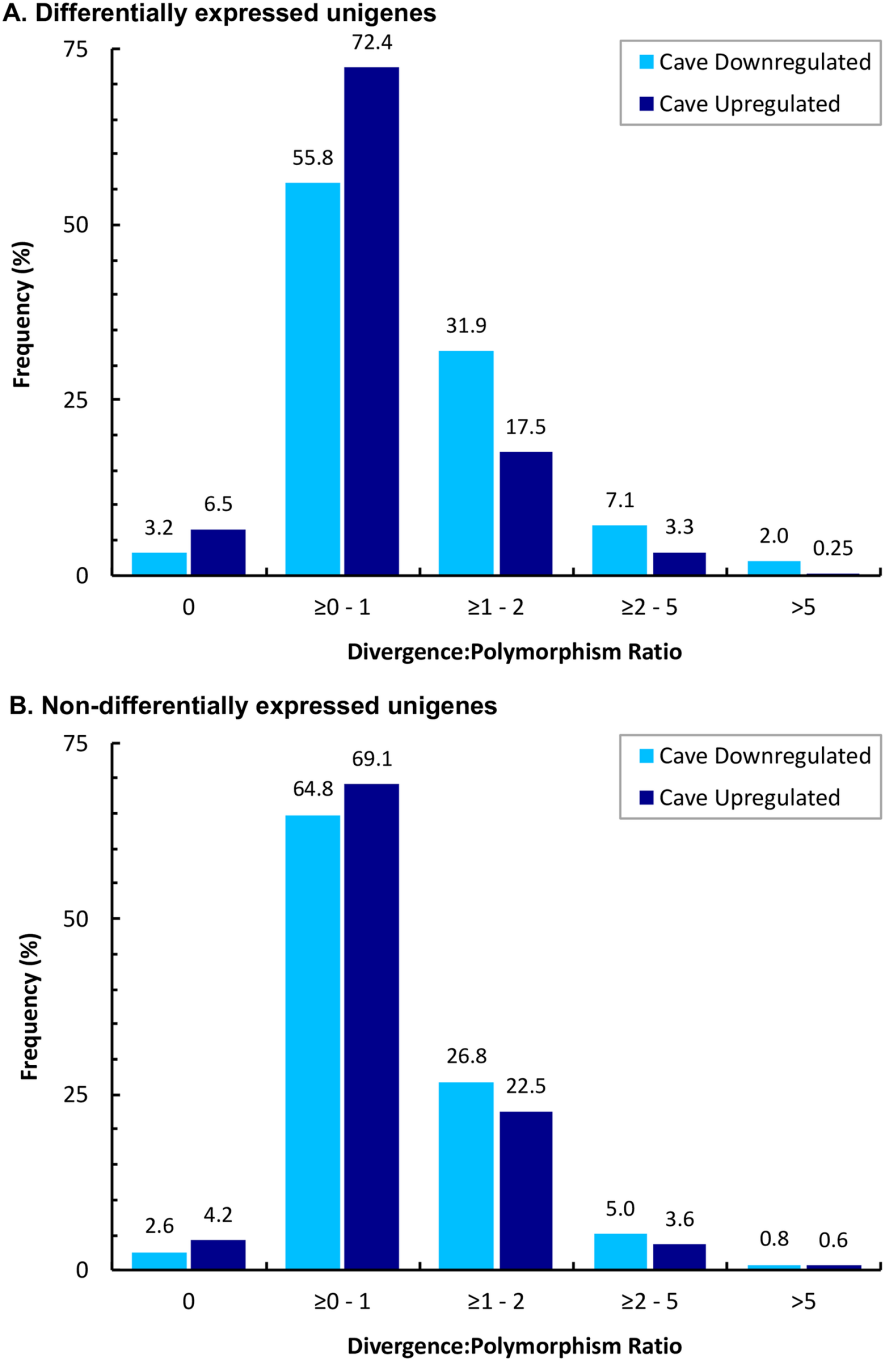
Frequency distributions of cave downregulated and cave upregulated unigenes grouped into five categories of divergence to polymorphism ratios, where low ratios (≤ 1) most likely corresponding to unigenes subject to purifying selection, and high ratios (>1) most likely corresponding to unigenes subject to positive selection. A. The frequency distributions of the 2,722 differentially expressed (*P* < 0.05) cave downregulated (dark blue) and cave upregulated (light blue) unigenes were significantly different (*P* < 10^−28^, 2×5 contingency test), with cave downregulated genes having a greater proportion of high divergence to polymorphism unigenes. B. Frequency distributions of the non-differentially expressed unigenes were much more similar between the cave downregulated and cave upregulated sets.

If positive selection acted on the CD transcripts in the OC population then, on average, those transcripts should exhibit a higher degree of sequence divergence from those of the WS population than the CU transcripts. In other words, there should be an enrichment of highly diverged CD transcripts. A total of 2,722 unigenes were significantly differentially expressed (raw *P* < 0.05), 1,622 of which were CU and 1,100 of which were CD. For each of these transcripts, the average nucleotide diversities within the OC and WS populations were calculated, as well as the average nucleotide sequence divergence between the OC and WS populations. The ratio of nucleotide sequence divergence to the sum of OC and WS nucleotide diversity (i.e., polymorphism) was used to quantify transcripts with very high (>5), high (≥2–5), intermediate (≥1–2), low (≥0–1), or zero (0) value ratios of divergence to polymorphism (Fig 5A). The frequency distributions of divergence to polymorphism ratios were significantly different between the CD and CU transcripts (*P* < 10^−28^, 2×5 contingency test). There was an eightfold enrichment of very highly diverged CD transcripts (2%) over CU transcripts (0.25%), and an over twofold enrichment of highly diverged CD transcripts (7.1%) over CU transcripts (3.3%), consistent with what would be expected if the CD transcripts were subject to positive selection. Conversely, CD transcripts had lower proportions of low or zero divergence transcripts in comparison with CU transcripts (55.8% CD versus 72.4% CU, 3.2% CD versus 6.5% CU, respectively). When considering the unigenes that were not significantly differentially expressed (raw *P* > 0.05), the frequencies of the very highly diverged and highly diverged classes in the CD and CU transcripts were similar (Fig 5B). For the very highly diverged class the frequencies were 0.8% CD versus 0.6% CU, and for the highly diverged class the frequencies were 5.0% CD versus 3.6% CU, and differences among the remaining three classes were even more muted.

### Excess of fixed premature termination codons in the OC population

Of the 8,134 unigenes with BLAST hits, a total of six transcripts contained fixed premature termination codons (PTCs) in one population. Five of these fixed PTCs were found in the OC population, and one fixed PTC was found in the WS population. In the OC population these PTC-containing transcripts are homologs of the following genes: 1) photolyase (codon 264 of 423 aligned codons, OC_RPM_ = 0.21×WS_RPM_, *P*<0.01), a light-dependent enzyme that repairs UV-induced DNA damage, 2) chymotrypsin C (codon 265 of 294 aligned codons, OC_RPM_ = 2.24×WS_RPM_, ns), a serine protease that functions to degrade the digestive protein trypsin, 3) spore coat protein SP65 (codon 50 of 112 aligned codons, OC_RPM_ = 0.90×WS_RPM_, ns), 4) solute carrier family 13 member 5 (codon 133 of 167 aligned codons, OC_RPM_ = 0.22×WS_RPM_, P<0.05), high-affinity sodium/citrate cotransporter that mediates citrate entry into cells, and 5) T-complex protein 1 subunit delta (codon 526 of 540 aligned codons, OC_RPM_ = 0.88×WS_RPM_, ns), a molecular chaperone that assists in protein folding. The sole transcript with fixed PTCs in the WS population is a homolog of protein-methionine sulfoxide oxidase MICAL2 (codons 16, 116, 138, and 142 of 643 aligned codons, OC_RPM_ = 1.02× WS_RPM_, ns), a nuclear monooxygenase that depolymerizes F-actin and regulates serum response factor signaling. In addition to these fixed PTCs, a number of unigenes contained PTCs in at least two individuals of either the OC (19 transcripts) or WS population (16 transcripts), or in both the OC and WS populations (5 transcripts).

### Relaxed selection on photolyase in the OC population

Because the downregulation and relaxed functional constraint of photolyase in the OC population makes evolutionary sense given the absence of UV and visible light in the cave environment, an additional ten individuals each from the OC and WS populations were collected, assayed for expression using qRT-PCR, and sequenced to validate the RNA-seq results and to determine if the PTC remained ‘fixed’ in the OC population. The qRT-PCR results confirmed that photolyase expression is downregulated in OC population, although the level of downregulation (0.67×, P<0.05) was less pronounced than that observed from RNA-seq (0.21×). All 10 individuals from the OC population were homozygous for the PTC (TAA), whereas all 10 individuals from the WS population were homozygous for the TAC tyrosine codon. Due to the complete absence of UV-light in the subterranean environment, a specific mechanism to repair UV-induced pyrimidine dimers may not be required in the OC population, and any mutations which negatively affect the function of the repair enzyme photolyase would be subject to relaxed selection. Interestingly, in preliminary experiments individuals from the OC population do appear to be less tolerant of UV exposure in the lab, suggesting that the photoreactivation repair mechanism is compromised due to the PTC in the photolyase gene of OC individuals (unpublished data). Photolyase may also function as a light-dependent circadian clock protein [41]. In that case it is also expected that the photolyase gene would be subject to relaxed selection in the OC population. To our knowledge this is the first published report of pseudogenization of a light-dependent DNA repair gene in a cave population. We are in the process of pursuing this interesting finding in greater detail, including the question of whether the apparent downregulation of photolyase is a consequence of nonsense-mediated decay or reduced transcription. Photoreactivation is an ancient repair mechanism, present in Eubacteria, Archaea, and Eukaryotes [42]. The photolyase gene has been lost in multiple lineages in all three groups including, notably, the placental mammals, which are completely dependent on the alternative pathway of nucleotide excision repair (NER) to remove pyrimidine dimers. This dependence on a single repair pathway potentially renders placental mammals, and other groups in which photolyase has been lost, more sensitive to UV-induced DNA damage [43, 44]. Lineages that have lost the photoreactivation pathway may be subject to higher genomic mutation rates as a consequence of their reduced ability to repair DNA damage [45], thus the fitness consequences of photolyase loss may be profound.

It is interesting that another transcript related to repair of UV-damaged DNA, a homolog of human DNA repair endonuclease XPG (*ERCC5*), was also downregulated (0.69×, P<0.05) in the OC population. *ERCC5* is a component of the NER pathway, and mutations in *ERCC5* can cause *Xeroderma pigmentosum*, a genetic condition in which individuals are extremely sensitive to UV radiation and are highly prone to skin cancers, including malignant melanomas [46]. In addition to its reduced expression, a total of four nonsynonymous polymorphisms were present in the OC population *ERCC5* transcript, whereas the WS population harbored only a single nonsynonymous polymorphism in *ERCC5*, indicating that this gene is also under relaxed selection in the OC population. Therefore, it is reasonable to expect that, as has been shown to be the case for genes related to vision, future research may demonstrate that downregulation and relaxed selection of genes functioning in the repair of UV-induced DNA damage is a general feature of subterranean organisms.

## Conclusions

This study has significantly enhanced our understanding of expression and sequence variation within and between a pair of cave and surface sister populations of the amphipod crustacean *G*. *minus*. When comparing the expression levels of individual transcripts in the cave and surface populations, we found an enrichment of upregulated transcripts in the cave population. The bias toward upregulation in the cave population was even more pronounced for transcripts that were differentially regulated. It is possible that the global higher expression levels in the cave compared to surface populations reflected a difference in response to the same post collection treatment based on divergent adaptation to the different habitats. In general, significantly downregulated transcripts in the cave population appear to have been subject to positive selection rather than relaxed or purifying selection. Consistent with previous studies which have compared the transcriptomes of subterranean and surface populations and/or species, several transcripts involved in the visual pathway were downregulated in the cave population. However, we also identified a novel pathway that might be influenced by relaxed selection in subterranean species and populations: photoreactivation, a highly conserved light-dependent process that functions to repair UV-induced DNA damage. As there are several pairs of cave and surface sister populations in *G*. *minus* that remain to be characterized at the transcriptome-wide level, it will be interesting to determine whether these findings are a general feature, or whether they are specific to the pair of populations examined in the present study.
